# Hydrogen competition between a gas reservoir community indicates available CO_2_ as main limiting factor

**DOI:** 10.1093/femsle/fnag031

**Published:** 2026-03-24

**Authors:** Nicole Dopffel, Larissa Compassi, Ben Heydolph, Abduljelil Kedir, Biwen Annie An-Stepec, Johann Badstöber, Tzvetanka Boiadjieva-Scherzer

**Affiliations:** NORCE Research AS, 5008 Bergen, Norway; OMV E&P GmbH, 1020 Vienna, Austria; NORCE Research AS, 5008 Bergen, Norway; NORCE Research AS, 5008 Bergen, Norway; NORCE Research AS, 5008 Bergen, Norway; OMV E&P GmbH, 1020 Vienna, Austria; OMV E&P GmbH, 1020 Vienna, Austria

**Keywords:** H_2_ underground storage, H_2_ consumption, H_2_ conversion, sulphate reducing microbes, methanogenesis, acetogenesis

## Abstract

Hydrogen (H_2_) underground storage in salt caverns or depleted gas reservoirs has been proposed as an economically viable option for storing large volumes of H_2_ to overcome seasonal fluctuations in energy production/demand. However, subsurface reservoirs often contain microbial communities that can consume H_2_ as an electron donor over time. To study the possible activity and H_2_ consumption within a reservoir, we sampled brine from a depleted gas field in Austria. Initial DNA community sequencing showed that the brine contained a diverse microbial community including sulfur-cycling microbes, methanogens, acetogens and general fermenters. In batch cultivation tests, we exposed the brine to different ratios of H_2_/CO_2_ (10, 40, and 95% H_2_ with 3–7% CO_2_). H_2_ was consumed in all cases, and we observed initial H_2_S formation, followed by what appeared to be simultaneous methanogenesis and acetogenesis. Activity ceased when CO_2_/dissolved carbonate was depleted. H_2_ consumption restarted as soon as CO_2_ was resupplied. Addition of original rock material, which is rich in carbonate minerals (26–50 Ma.-% Calcite), boosted activity. The results show that for a mixed H_2_-consuming community, the limiting factor is CO_2_ when H_2_ is in excess. This will have direct effects on pH and the competing activity of methanogens and acetogens.

## Teaser

Microbial communities from a depleted gas reservoir rapidly consumed hydrogen, with activity driven by COâ‚ availability and enhanced by carbonate-rich rock, revealing competition effects of methanogenesis and acetogenesis during underground hydrogen storage.

## Introduction

Hydrogen (H_2_) is becoming a key part of the clean energy transition. It can be produced using renewable electricity, stored, used as a feedstock for certain industries, and even help stabilize the electricity grid (Koohi-Fayegh and Rosen 2020). By storing high volumes of excess H_2_, it can be used later during periods of high energy demand or low renewable output and allows H_2_ to be used as a strategic reserve. For this scenario to succeed, relevant H_2_ volumes need to be stored for a certain amount of time (weeks to months). The first step will be H_2_ storage in solution mined salt caverns. However, salt caverns are restricted to certain geological areas (mostly to be found in Germany, Netherlands, France, and UK) and have a limited volume of up to 500.000 m^3^ (Dopffel et al. 2025). Storing volumes beyond that can only be achieved using porous reservoirs such as depleted gas fields (Heinemann et al. 2021, Bade et al. 2024). These fields offer a scalable way to store large volumes of H_2_ underground. As they have overlying layers of impermeable caprock, preventing the gas from escaping, and they also have already developed infrastructure, depleted gas fields are promising large-scale sites for long-term seasonal storage (Tarkowski and Uliasz-Misiak 2022).

However, careful site selection and monitoring are crucial to ensure the H_2_ remains stable and does not interact or react with the surrounding geology or microbes. Many different types of microorganisms can consume H_2_ during their activity and growth, thereby affecting H_2_ quantity and quality (Gregory et al. 2019, Ranchou-Peyruse 2024). The most relevant subsurface microbial groups, which can consume H_2_ are sulfate-reducing microbes, methanogens, and acetogens. In addition to reducing the amount of usable H_2_, those microbes can also produce byproducts such as the toxic H_2_S (by sulfate-reducers), methane (by methanogens), or acetate (by acetogens). Moreover, microbial processes can alter the chemical composition of reservoir brine and interact with reservoir rock minerals, potentially affecting its long-term reservoir suitability for repeated storage cycles (Dopffel et al. 2021, Haddad et al. 2022). Because of these risks, understanding the microbial ecosystem of a storage site is essential before using it for H_2_ storage, and in some cases, measures may be taken to control or limit microbial activity.

Several H_2_ storage studies and field trials have investigated microbial activity in depleted gas fields, including the Underground Sun Storage project in Austria, HyChico in Argentina, HyStorage in Germany (Pérez et al. 2016, RAG 2017), and they all observed microbial H_2_ conversion but at different rates and different main metabolisms, ranging across all three mentioned main groups. These sites were inherently different from their chemical, physical, and mineralogical characteristics. Findings cannot be transferred easily to other sites, especially since mineralogy might play an important role. Microbes can interact with minerals in various ways, and they use them as electron donors and acceptors, which leads to dissolution or precipitation (Banfield and Nealson 2018). Particularly, carbonate minerals are of crucial importance as they are known to be usable as a CO_2_ source for different microbial groups (Ranchou-Peyruse et al. 2024, Ranchou-Peyruse et al. 2025). Methanogens have been shown to be directly stimulated by mineral presence (Kato et al. 2012). As minerals also have a direct effect on the geochemistry, the interactions with microbes will directly affect the brine and therefore the reservoir properties (Haddad et al. 2022). It has been shown that intricate interactions can occur when a complex subsurface community is exposed to H_2_ over time with the co-occurrence of CO_2_, leading to the assumption that the carbon source is a main driving factor (Mura et al. 2025). Still, many aspects of the syntrophic or competing interactions are poorly investigated.

As knowledge of the microbial processes involved in underground H_2_ storage remains limited, and depleted reservoirs can differ dramatically in their microbial characteristics, we focused our investigation on a specific reservoir in Austria. By conducting a detailed site-specific study, we aimed to gain insights into the local microbial community, its interactions with H_2_, limiting factors, and the implications for long-term storage stability and gas integrity. This approach not only enhances our understanding of subsurface microbial dynamics but also contributes to the development of more reliable and efficient H_2_ storage strategies in similar geological settings

## Methods

### Sampling and physical parameters

Samples were taken from a relevant reservoir located in Austria in April 2023. The reservoir parameters and mineralogical information are given in Table 1 and Supplemental Table 1. The brine is of freshwater quality, and water chemical composition confirmed that the samples are indeed reservoir formation water. However, atypical for the Eocene horizon, rather corresponds to the Upper Cretaceous formation. Reservoir brine samples were collected directly at the wellhead into sterile and argon filled glass bottles to preserve anoxic conditions and afterwards immediately shipped to the laboratory. Physical parameters of the reservoir were given by the operator of the field. Mineralogical information about the utilized rock material was obtained via XRD.

**Table 1:** Geological and chemical information from the sampled field.

General information	
Perforation [m]	619.0–625.0
Horizon [m]	560–90
Formation	Eocene
Perforation at [m]	619–625
Permeability [mD]	0.2
Porosity [%]	4.62
Average mineralogical composition [mass %]	
Quartz	38.6
Calcite	38.4
K-Fsp.	19.0
Plag	1.8
Dolomite	0.5
Clay Tot + Mica	0.7
Ankerite	0.7
Pyrite	0.3
Well pressure *at the time of water sampling*	∼10 bar
Reservoir Temperature	41°C
Brine information	
pH	7.8 ± 0.1
Salinity (w/w) %	0.43 ± 0.01
Sulphate [mg/L]	62.4
Volatile fatty acids [mg/L]	Acetate: 295.6 ± 3.2

### Analysis

pH was measured on an open membrane type pH-meter (LAQUA twin pH-22 compact, Horiba, Japan). Salinity (wt/wt) was measured with a standard pocket salt meter (ATAGO PAL-1 refractometer, ATAGO co., LTD, Japan) against a reference. Volatile fatty acids, especially acetic acid and formic acid, were analyzed by using liquid chromatography on an Agilent 1260II UHPLC equipped with a flexible pump, autosampler, 1260 RI, and 1260 DA HS detectors. All analytes were identified and quantified based on their respective reference standard calibration curves. Gas composition was measured with a micro gas chromatography (microGC) 490 (Agilent) by directly measuring the gas in the headspace of the serum bottles.

### Microbial cultivation and enrichments

To enrich possible aerobic species, 100 µL of the brine was streaked on standard LB plates. Incubation of the plates was done at 30°C. For anaerobic cultivation, 25 mL of brine was filled in individual sterile bottles (total volume 58.35 mL) containing different gas mixtures in the headspace. All bottles containing H_2_ were stored upside down to minimize loss of H_2_ due to diffusion through the rubber stopper. Still, we observed diffusion through the stoppers, especially over longer incubation periods. Anoxic distilled water in bottles with the same stoppers was always taken along as diffusion controls, and the loss through the stoppers was always subtracted from the microbial bottles. Different gas mixtures used were as follows: 93% H_2_ + 7% CO_2_ or 40% H_2_ + 2% CO_2_ or 10% H_2_ + 2% CO_2_. The higher CO_2_ content in the 93% H_2_ bottles is due to a handling error; however, this does not affect the overall interpretation. The rest of the gas was either N_2_ or CH_4_. Controls were bottles with the addition of only N_2_. Another set of bottles contained brine with added acetate (20 mM) and 0.04% yeast extract to boost microbial activity. At different time points, CO_2_ and/or H_2_ were refilled in the bottles. Sterile controls were autoclaved brine. For another set of experiments, original crushed rock material was provided by the reservoir operator. The rock was obtained in 1969 and has been stored dry and in air. The rock was homogenized in the lab and not pre-treated in any way initially. 10% (around 2.5 g) or 50% (around 12 g) was filled into sterile bottles and flushed with N_2_ for several minutes before adding the reservoir brine. Brine was added to these minerals on top until a volume of 25 mL was reached. Furthermore, 60 mg/L sulphate was added because shipping and storage of the brine for ∼4 weeks caused sulphate-reducing activity and likely led to the loss of all sulphate in the brine. This brine was kept with a 98% N_2_ + 2% CO_2_ headspace for 4 days to let the brine and rock equilibrate. pH measurement was performed. As sterile controls, brine was autoclaved to kill the microorganisms and added to the untreated rock. Gas headspaces were filled with 98% H_2_ + 2% CO_2_. Additionally, some rocks were autoclaved three times on three consecutive days. Brine and gas were added as described before. All incubations were at 41°C upside down without shaking.

### Sampling and calculations

Pressure measurements, gas analysis, and liquid sampling were performed in regular intervals during the incubation. Pressure in the bottles was measured using a pressure sensor from Sensortechnics 0–3 barg Press D/C 2916 with an individual set-up for direct measurement of the headspace of serum bottles. One mL of liquid was withdrawn for pH and HPLC determination. To calculate the amount of H_2_ in the bottles, the ideal gas law was used, as described before (Dopffel et al. 2023). Pressure was always measured at the beginning and the end of a sampling. This enables us to calculate the loss of H_2_ due to sampling. This sampling loss was subtracted from the H_2_ values at the end to ensure that only microbial H_2_ consumption is reflected.

### Community analysis

For 16S community analysis based on DNA, 20 mL of brine were filtered a 0.2-µm membrane filter immediately upon arrival at the laboratory (Černá et al. 2025). They were immediately frozen at −20°C. For determining the community of the active enrichments, we isolated DNA from 1 to 5 mL of the sample, which was withdrawn using syringes and centrifuged for 20 min at 13 000 rpm. The pellet was frozen at −20°C for storage. DNA from the filters and samples containing soil was extracted from the filters using the DNeasy Power Soil Kit (Qiagen). DNA from liquid only was isolated using the DNeasy Blood & Tissue Kit (Qiagen) following manufacturer’s instructions. DNA concentration was determined via Qubit dsDNA High Sensitivity assay (Invitrogen). The bacterial and archaeal community was obtained via Illumina Nextera two-step libraries with the V4 region (515F: NNNNNGTGYCAGCMGCCGCGGTAA, 806R: NNNNNGGACTACNVGGGTWTCTAAT) of the *16S rRNA* gene on the Illumina MiSeq platform with 2×250 bp reads performed by Microsynth (Switzerland). Bioinformatic analysis contained locus specific adaptor sequences (cutadapt), merging of forward and reverse reads, quality filtering (Usearch), ASV building and chimera removal (Usearch) and 16S taxonomy assignment based on the Silva138 database. The taxonomic diversity index can be found in the Supplemental Table 2. All ASVs are deposited online with the accession numbers PX499662-PX499685.

### Cell enumeration

The initial number of total cells, as well as the quantification of different metabolic groups, was analyzed using the Genecount® RT-qPCR workflow from Luminultra. At the sampling site, 50-mL water brine were filtered over a 0.2-µm filter, which was subsequently preserved in Buffer A Solution from the Luminultra kit and stored at 4°C. In the laboratory, the preserved filter was processed by vortex mixing (V-32, Grant Instruments), heating for 5 min at 50°C (myBlock™ HL Mini Dry Bath, Benchmark), and centrifugation for 2 min at 10 000 rpm (MagFuge®, Heathrow Scientific). DNA extraction was carried out using the Genecount® Purify qKit (Luminultra) with magnetic bead purification, automated on the GeneCount® E-32 system. Real-Time quantitative-PCR was performed using Genecount® qPCR Test Kits targeting total archaea, total bacteria, methanogens, sulfate-reducing bacteria, sulfur-oxidizing bacteria, and iron-reducing bacteria on the Genecount® Q-96 platform. Each run included positive and negative controls. The thermal cycling protocol consisted of an initial denaturation at 95°C for 3 min, followed by 40 cycles of 95°C for 20 s and 60°C for 45 s. Fluorescence detection was conducted using FAM and SYBR channels.

## Results

### Field characteristics and field microbiology

Analysis of the reservoir brine shows a low-salinity, low-sulfate brine with a circumneutral pH around 7.8 (see Table 1). Cell number analysis detected both Bacteria (6.34E + 07 cells/mL) and Archaea (8.76E + 05 cells/mL) being present with significant numbers of sulfate- (1.95E + 05 cells/mL), and iron-reducers (1.09E + 07 cells/mL) and methanogens (7.98E + 05 cells/mL) (Table 2), all metabolic groups known to be able to consume H_2_. The brine contains a background acetate value of around 300 mg/L of unknown origin. This acetate could originate from drilling mud (Haddad et al. 2022) or could be product from microbial fermentation. We did not observe any growth on aerobic LB agar plates, indicating that the brine did not contain any aerobic heterotrophs. Gas analysis of the sample bottles showed CO_2_ concentrations of around 2% in the headspace, which shows that the brine contains dissolved carbonate, which outgassed over time. To stabilize the pH, at least 2% CO_2_ has to be added to all bottles.

**Table 2:** Initial cell number quantification by qPCR of different metabolic groups.

Group/Measurement	Unit	Calculated cell number
Total archaea	cells/mL	8.76E + 05
Total bacteria	cells/mL	6.34E + 07
Total methanogens	cells/mL	7.98E + 05
Sulfate-reducing microbes	cells/mL	1.95E + 05
Sulfur-oxidizing bacteria	cells/mL	1.59E + 04
Iron reducing bacteria	cells/mL	1.09E + 07

**Figure 1:**
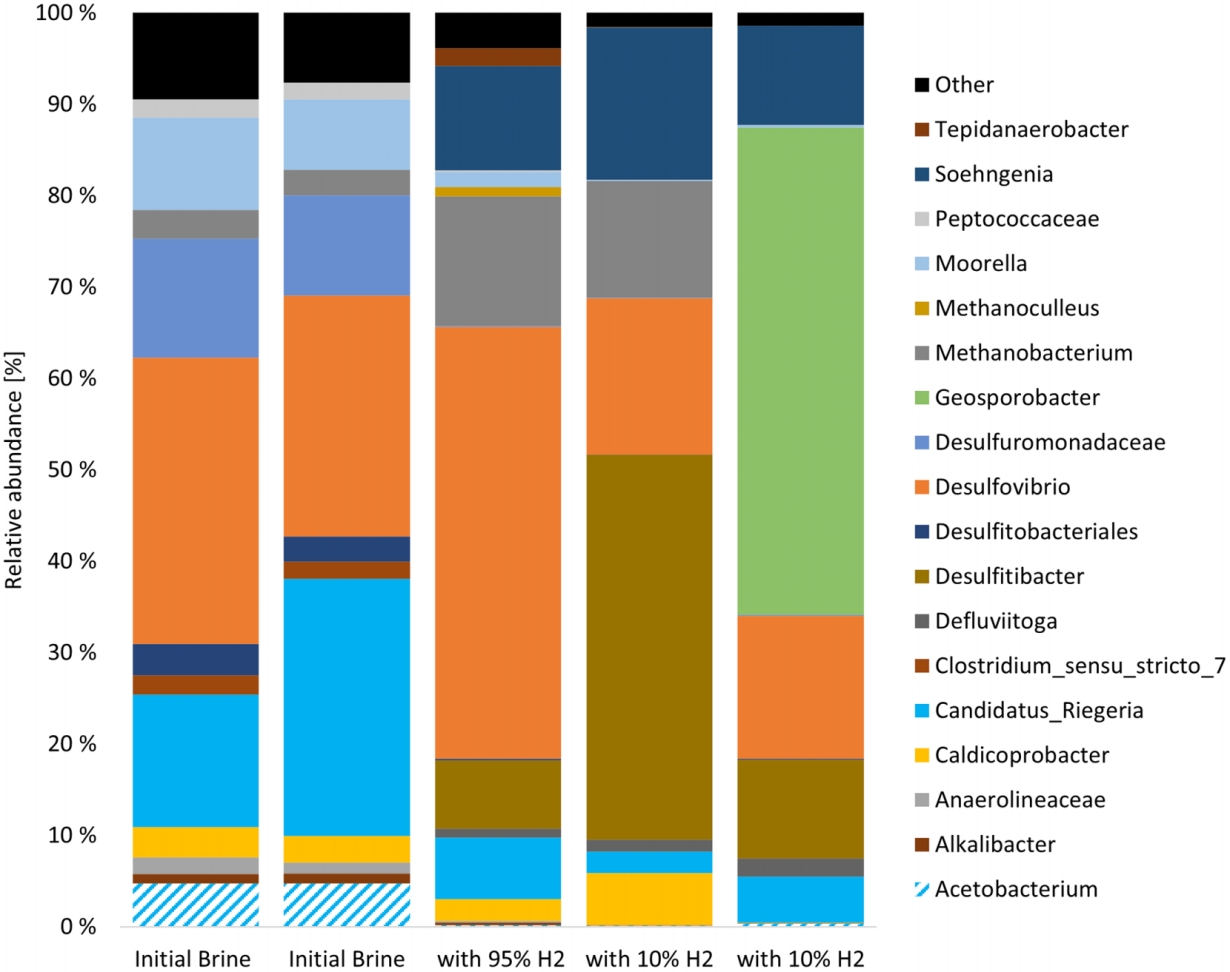
Microbial community composition based on 16S rDNA data at genus or family level with minimum 1% abundance.

The initial community structure of the initial filters shows a general low taxonomic diversity and a high relative abundance of sulfur cycling groups, including sulphate reducers and sulfur oxidizers (Fig. 1), which fits the qPCR. Acetogenic bacteria are represented by *Acetobacterium* and *Moorella*. Methanogenic archaea are present mainly with members of *Methanobacterium*. A smaller fraction of fermenting microbes, alkane degraders like *Anaerolineaceae* could also be detected. Overall, the community is a typical reservoir/subsurface community with a surprisingly high portion of sulfur-cycling organisms considering the low amounts of sulfate in the brine. It cannot be ruled out that other sulfur species play a role, including thiosulfate, or that the microbes are active with a different metabolic function. The present microbial groups already indicate a very high risk of H_2_ consumption in this brine, as all three major risk groups (sulfate reducers, acetogens, and methanogens) are represented.

### Microbial consumption of H_2_ and CO_2_

**Figure 2:**
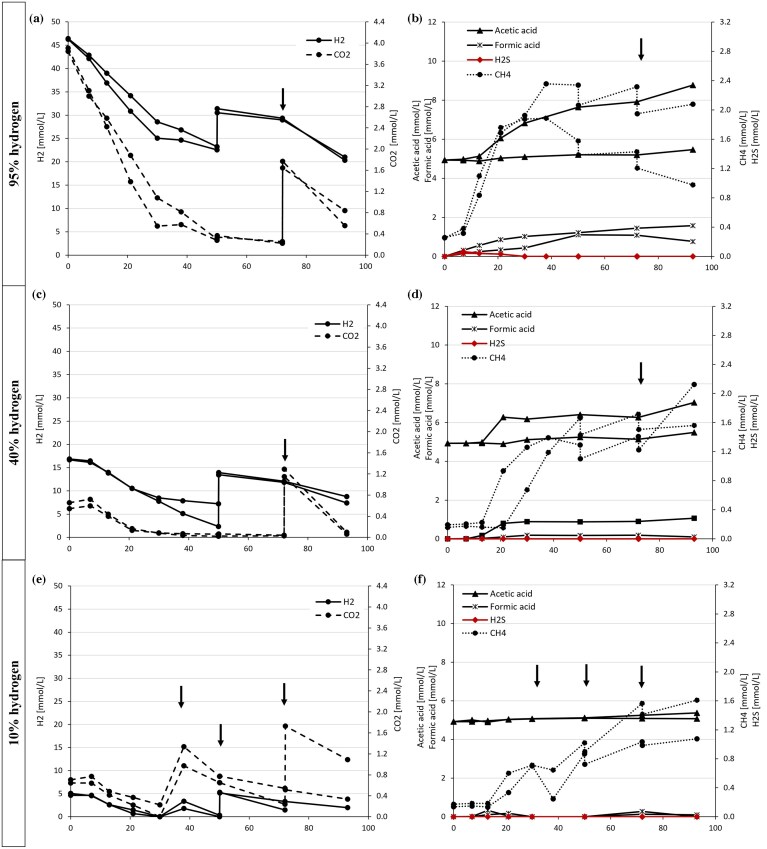
H_2_ (●solid line) and CO_2_ (●dashed lined) consumption over time (left side panels). H_2_S (♦ solid line), CH_4_ (● dotted line), acetic acid (▴solid line), and formic acid (x solid line) production over time (right side panels) for the bottles amended with 95% H_2_ (a and b), 40% H_2_ (c and d), and 10% H_2_ (e and f). All units are represented in mmol/L. Values from duplicate bottles are represented with the same line type and not averaged but shown individually. Arrows indicate the addition of new gas, either H_2_, CO_2_ , or both, in the headspace.

We supplied different H_2_ concentrations to the headspace of the bottles (10, 40, and 95%) reflecting the possibility of different storage scenarios for the field (ranging from low to high purity H_2_ storage). In all cases, we observed H_2_ consumption (Fig. 2). Having N_2_ or CH_4_ as the remaining gas did not affect the results (data not shown).

In the 95% bottles containing around 7% CO_2_ (Fig. 1a and b), H_2_ consumption rates were highest (0.021–0.025 mmol/day) until H_2_ consumption slowed down or even stopped around day 30 and was minimal until day 50. Re-supply of H_2_ did not change activity, and almost no H_2_ was consumed until the addition of CO_2_ on day 72. This addition led to a resumed H_2_ consumption activity. We observed FeS precipitation and H_2_S production with a maximum value of 0.14% in the headspace on day 7, after which it declined, reaching 0% at day 30. Concurrently, the pH increased significantly from 7.4 to a maximum of 8.4 (Supplemental Table 3). At higher pH, H_2_S will be in its soluble form HS^-^, which explains the decrease in the gas phase. Methane was produced in both bottles after day 7. Acetate production was significant only in one of the duplicates, reaching a value up to 520 mg/L (Fig. 2b and Supplemental Table 4). Formate, as a precursor of acetate formation, could also be measured over time (Supplemental Table 5). Similarly, with 40% H_2_ + 2% CO_2_ in the headspace, we measured a decline in H_2_ with a concurrent increase in methane in both bottles and a significant increase in acetate in one bottle (Fig. 2c and d). On day 50, we re-supplied only H_2_, but no major consumption was observed within the next 20 days. After the addition of CO_2_, activity resumed. End pH was very high, reaching values over pH 9 (Supplemental Table 3). Acetate did increase up to 422 mg/L. In the 10% H_2_ bottles, all H_2_ was consumed starting on day 7 and finishing within 30 days with a maximum rate of 0.011 mmol/day (Fig. 2e and f). Re-feeding of 10% H_2_ plus CO_2_ to the headspace leads to continuous, somewhat faster H_2_ consumption within 20 days (max rate 0.015 mmol/day). A third addition of 10% H_2_ was also consumed but with a slower rate (max rate 0.067 mmol/day), which might be caused by recurrent increase in pH up to pH 8.5 (Supplemental Table 3). No H_2_S was measured in the headspace at any time, but methane was produced after day 14, continuously increasing until the end of the experiment. We measured a slight increase in acetate from 295 mg/L at day 0 to 305–322 mg/L after 96 days. The brine without hydrogen addition did not show significant pH changes, H_2_S, or methane production. We did, however, observe an increase in acetate from 295 up to 332 mg/L (Supplemental Table 4). This indicates that some background fermentation of organic components is responsible for some of the observed acetate increase.

DNA extraction from the brine samples proved to be difficult and after several attempts we were only able to obtain DNA and sequence information from three enrichment samples (Fig. 1 and Supplemental Table 10), including one bottle with high H_2_ content (relating to Fig. 2a and b) and two bottles with low H_2_ content (relating to Fig. 2e and f). The addition of H_2_, led to an increase in *Desulfitibacter, Methanobacterium*, and *Soehngenia*. With 95% H_2_ at the beginning of the experiment, there was a significant increase in *Desulfovibrio* relative abundance. The two bottles with 10% H_2_, showed different end communities with one bottle being dominated by *Desulfitibacter* and the other bottle being dominated by *Geosporobacter* (Fig. 1).

### Addition of original minerals

**Figure 3:**
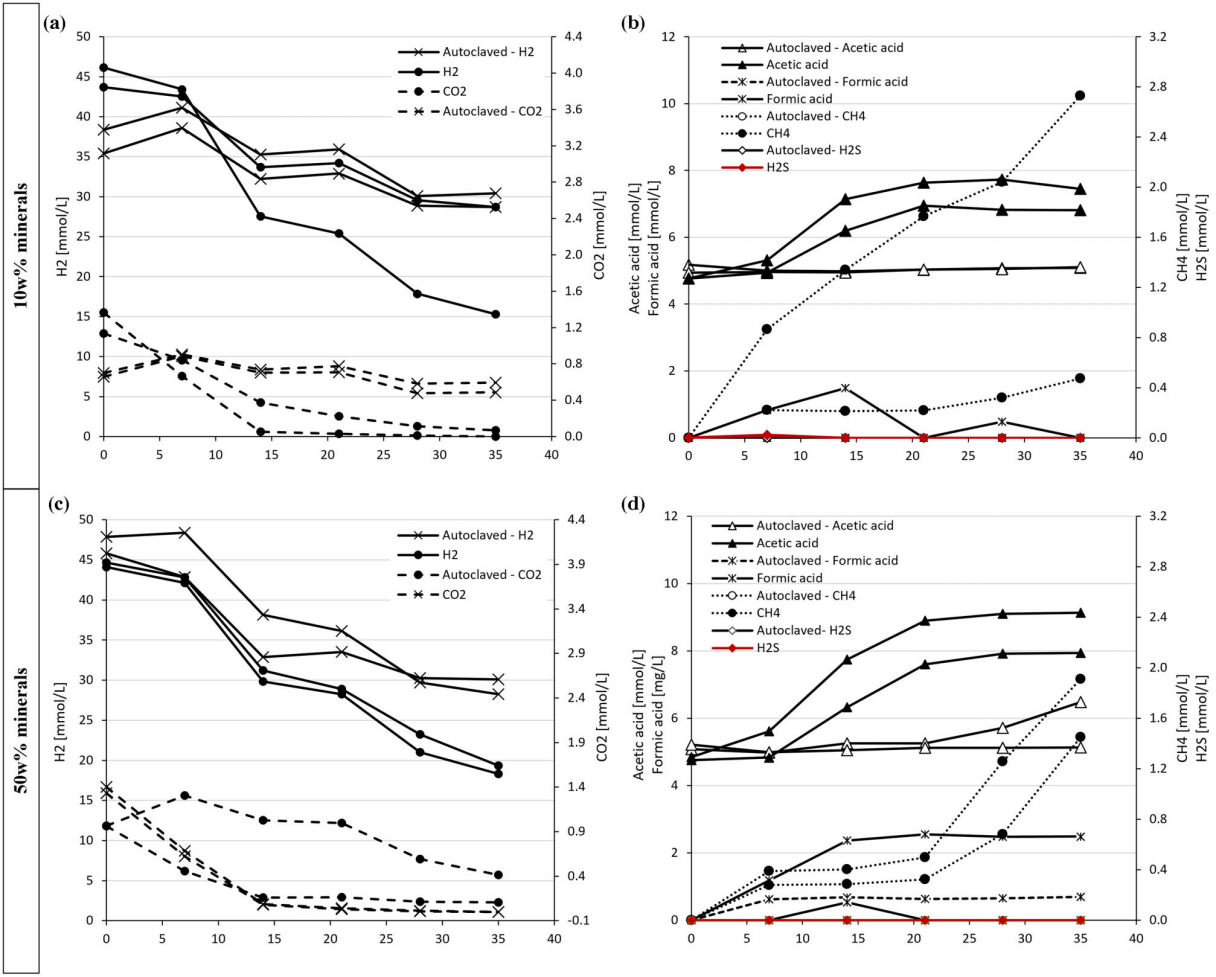
H_2_ (● solid line) and CO_2_ (● dashed lined) (x for autoclaved bottles) consumption over time in mmol (left side) and H_2_S (♦ solid line), CH_4_ (● dotted line), acetic acid (▴solid line, open symbols for autoclaved bottles), and formic acid (x solid line, dashed line for autoclaved bottles) production over time (right side) for the bottles amended with 10w% minerals (a and b), 50w% minerals (c and d). All units are represented in mmol/L. Values from duplicate bottles are not averaged but shown individually.

**Figure 4:**
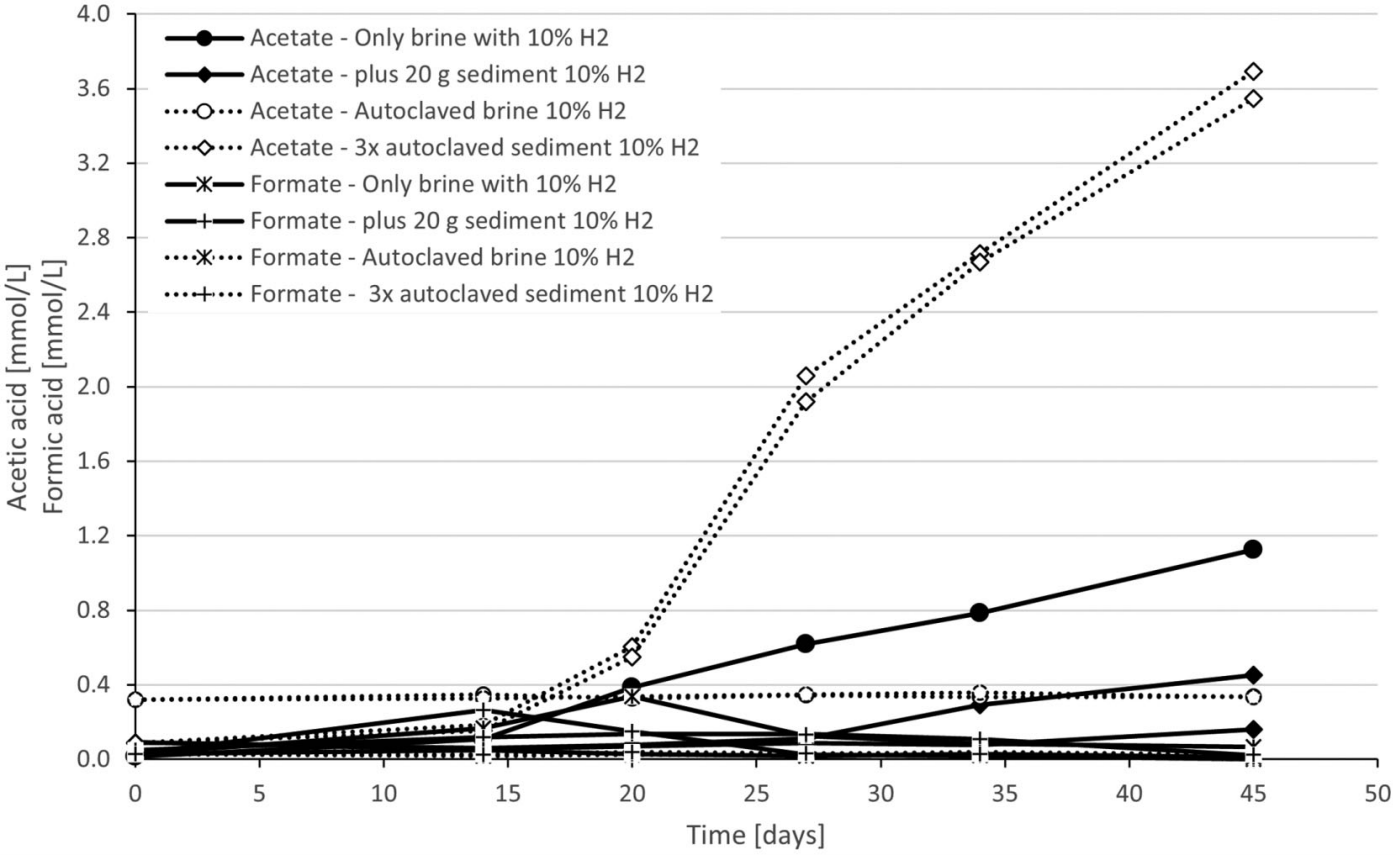
Acetic acid and formic acid content in mmol/L over time in enrichments with living reservoir brine with 10% H_2_ without (●, x solid line) or with untreated (♦, + solid line) 20 gram of rock material. Dashed lines indicate enrichments with autoclaved brine only (°, x) and with three times autoclaved rock with autoclaved brine (◊, +).

As the community is clearly limited by CO_2_, we tested whether original rock can be used as a carbonate source. XRD measurements from the reservoir rock do indeed contain carbonate minerals, especially significant amounts of calcite of an average of 38% (Supplemental Table 1). As it is known that minerals like calcite might also chemically react with H_2_ (Bensing et al. 2022), some brine was autoclaved as sterile control set-ups. Initially, the rock was not treated to avoid changing the mineralogy, and 2% CO_2_ was added to the headspace to stabilize the pH. With 10 w% of minerals (Fig. 3a and b), CO_2_ in the headspace was quickly consumed after 15 days, but CH_4_ and acetate production continued after that. H_2_S was observed, similar to the previous experiment, and the maximum pH value was pH 9.7 (Supplemental Table 6), indicating significant microbial activity. The rock itself carries residues of hydrocarbons, which might be an additional carbon source and activate fermentation. One bottle of the living set-ups showed much lower H_2_ consumption, but pH values still increased up to 8.9. The addition of 50 w% minerals had several interesting effects on the system (Fig. 3c and d). The amount of acetic acid produced was significantly higher (+ 190–257 mg/L compared to 123–161 mg/L in the 10 w% set-ups) (Supplemental Table 7), but less methane and no H_2_S could be detected. pH increased over pH 10. Indeed, we observed acetic acid formation and pH increase in the autoclaved controls, indicating insufficient removal of acetogenic spores. To test this hypothesis, we autoclaved the rock three times on three consecutive days in addition to autoclaving the brine. Again, we observed significant acetogenic growth (Fig. 4 and Supplemental Table 8), with much higher acetic acid accumulation compared to the non-autoclaved brine. Autoclaved brine showed no activity, meaning successful removal of methanogens and other microbes in the brine itself, whereas autoclaved rock samples showed both CO_2_ and H_2_ consumption over time (Supplemental Table 9). This indicates that the rock contains spores, which will start growing on the H_2_ as soon as it emerges in the brine. We were not able to extract DNA from the samples containing reservoir rock. Neither pure rock nor brine in addition to rock. This could be caused by inhibitors stemming from the rock, and more method establishment is needed to be able to identify the acetogenic spores, which are presumed attached to the rock surface.

## Discussion

### Reservoir properties indicate microbial risk for hydrogen conversion

The reservoir brine was sampled from a depleted porous gas reservoir, which is under investigation for a H_2_ storage trial. The reservoir itself is a depleted gas storage field with favourable parameters when it comes to tightness, location, and infrastructure. However, the field community data obtained based on DNA level shows many potential H_2_ consuming groups. Based on the current knowledge, the three main metabolic risk groups are sulfate-reducing microbes (e.g. *Desulfovibrio*), methanogens (e.g. *Methanobacterium*), and acetogens (e.g. *Moorella*), which are represented in the tested brine by at least one major family or more. But also, other potential H_2_-consuming groups like iron-reducers are detected, which can be part of an ongoing iron-cycling in reservoir. The overall presence was also confirmed via qPCR. The water composition is low in sulphate (62.4 mg/L; Table 1), which limits the activity of sulfate-reducing microbes. Still, we were surprised to see many sulfur-cycling microbial groups to be present. This indicates that other sulfur species might play a role, or sulfur is accessible inside the reservoir in the form of minerals like pyrite (see XRD data in Supplemental Table 1).

### Competing for hydrogen and CO_2_

When the pure brine was exposed to H_2_, rapid conversion took place, but here the overall amount of H_2_ present in the bottles influenced the resulting products. Only with high concentrations of H_2_ in the beginning, H_2_S was measurable in the headspace before disappearing again. This process drives an increase in pH, which will lead to dissolution of H_2_S in the brine that will react with the iron species in the brine forming iron sulfide. This reaction was reflected in the microbial community where the enrichment shows the dominance of the sulfate-reducing microorganism *Desulfovibrio*. With lower starting concentrations of H_2_, H_2_S was not observed. H_2_S production was at a maximum 0.13% (1342 ppm). The brine contains 62.4 mg/L sulfate, which is 0.6 mM. Based on the volume of the bottles, this would allow for 1.24% H_2_S if all H_2_S were to enter the gas phase (which it does not at neutral or alkaline pH). Also, 0.6-mM sulfate allows SRBs to consume 0.065 mmol H_2_. This shows that the major H_2_ loss is caused by methanogens and acetogens.

It could be seen that between the different set-ups, similar amounts of methane were produced between 0.11 and 0.07 mmol (average 0.086 mmol ± 0.015). This would mean that SRB and methanogens together consumed on average 0.15 mmol H_2_, and that the rest is consumed by acetogens. Microorganisms, which can utilize H_2_ as an electron donor, obtain different energy yields from the catalyzed reactions. The competition for H_2_ is influenced not only by thermodynamics but also by kinetics, substrate thresholds, microbial affinities and sulfate availability (Dar et al. 2008). It is thermodynamically more favourable that sulphate-reduction (high sulphate-availability) and methanogenesis occur prior to homoacetogenesis under standard conditions, but under low sulphate conditions, methanogenesis or acetogenesis may occur earlier or concurrently (Jackson and McInerney 2002, Dar et al. 2008). In our experiment, it was shown that SRB quickly converted the sulphate into H_2_S, which we were able to measure in the headspace. However, due to the low sulphate content in the brine, the H_2_S concentrations were relatively low and quickly decreased due to an increase in pH (Dopffel et al. 2023). Under standard conditions, methanogenesis has a higher energy yield compared to acetogenesis and is therefore thermodynamically favoured. Therefore, it was expected that acetogenesis starts only when methanogenesis stops, as it was described for an aquifer previously (Mura et al. 2025). However, we observed methane and acetate/formate production seemingly simultaneously, which is reflected in a scenario where sulphate is extremely limited and also indicates that both the methanogenic and acetogenic community responded quickly to the presence of H_2_ (Kakuk et al. 2021). Within a porous media, this could be explained by micro-niches where the groups are located in different areas, but this is unlikely in our well-mixed single bottle set-ups. Acetogenic activity was observed in all bottles independent of initial H_2_ concentration, but end concentrations were very different even in duplicate bottles (Table 3), indicating that even small changes in conditions or pure statistics will decide which product will accumulate. Another complicating aspect is that the acetate and formate concentrations are a result of a complex community activity, including formate/acetate-producers like fermenters and acetogens and formate/acetate consumers like sulfate- and iron-reducers or methanogens (which can often utilize formate and sometimes acetate (Costa and Leigh 2014)). Certain strains of sulfate-reducing microorganisms are also capable of fermentation in the presence of a syntrophic partner, such as methanogens (Plugge et al. 2011). Therefore, this cryptic cycle of acetate/formate production and use makes interpretation difficult.

**Table 3:** All key end values for the experiments.

	Available H_2_ [mmol]	Consumed H_2_ [mmol]	Available CO_2_ [mmol]	Consumed CO_2_ from headspace [mmol]	Ratio H_2_/CO_2_	CH_4_ produced [mmol]	Ratio H_2_/CH_4_	H_2_S produced [max%]	Acetic acid produced [mg/L]	Formic acid produced [mg/L]	End pH
+95% H_2_	1.89	0.87	0.19	0.17	5.2	0.11	7.9	0.13	32.0	35.1	8.1
+95% H_2_	1.88	0.83	0.18	0.15	5.5	0.07	11.4	0.13	230.7	72.4	7.9
+ 40% H_2_	0.81	0.49	0.07	0.06	7.8	0.10	5.1	0.00	33.9	4.6	8.8
+ 40% H_2_	1.01	0.64	0.07	0.06	10.2	0.07	8.7	0.00	126.5	48.8	8.8
+ 10% H_2_	0.51	0.44	0.07	0.06	7.5	0.07	6.2	0.00	26.8	4.7	8.4
+ 10% H_2_	0.56	0.56	0.14	0.10	5.7	0.09	5.9	0.00	9.1	0.0	7.9
Autoclaved + 10% rock	1.28	0.05	0.03	0.00		0.00		0.00	0.0	0.0	8.3
Autoclaved + 10% rock	1.18	0.01	0.03	0.00		0.00		0.00	9.7	0.0	8.2
+ 10% rock + 95% H_2_	1.54	0.86	0.05	0.04	22.3	0.10	9.9	0.03	161.3	21.9	9.7
+ 10% rock + 95% H_2_	1.46	0.29	0.04	0.03	10.2	0.02	24.0	0.04	123.0	68.2	8.9
Autoclaved + 50% rock + 95% H2	1.60	0.44	0.04	0.01	44.0	0.00		0.00	83.9	0.0	8.2
Autoclaved + 50% rock + 95% H2	1.53	0.31	0.03	0.02	14.6	0.00		0.00	0.0	31.9	8.8
+ 50% rock + 95% H_2_	1.47	0.68	0.05	0.04	17.0	0.05	15.2	0.00	190.9	117.6	9.9
+ 50% rock + 95% H_2_	1.49	0.66	0.05	0.04	17.6	0.07	11.3	0.00	257.8	24.4	10.1

The identified microbial community from the H_2_-consumption experiments indicated several key players. With a high initial H_2_ concentration, we have a high abundance of *Desulfovibrio*, reflecting also the formation of H_2_S. In the two bottles with 10% initial H_2_, either sulfite-reducing *Desulfitibacter* or iron-reducing *Geosporobacter* was significantly enriched. *Geosporobacter* species are anaerobic, alkaliphilic or alkali–tolerant, heterotrophic iron–reducing Firmicutes commonly found in subsurface and oil–impacted environments (Hong et al. 2015). *Desulfitibacter* species are also anaerobic, alkalitolerant, sulfite–reducing bacteria which reduce sulfite, thiosulfate, elemental sulfur, nitrate, and nitrite, but not sulfate or Fe(III) (Nielsen et al. 2006). The closest relatives to our detected ASVs are both described as alkaliphilic or alkali-tolerant. (Nielsen et al. 2006, Hong et al. 2015). Thus, they might have been favoured in the end community due to the increased final pH. We also observed an increase in sequences of *Soehngenia*, which is a strictly anaerobic, fermentative bacteria commonly recovered from subsurface petroleum reservoirs and it can be associated with methanogenic microbes (Nazina et al. 2020). How the observed 16S community translates into predicted functions (Belcour et al. 2025), should be investigated in future studies.

### Influence of rock and minerals

One major driving factor in our bottle experiments was that there was no limitation of H_2_, making our system and also the hydrogen storage sites a quite unique ecosystem on Earth. Whereas in most environments, H_2_ is transient and only present in nmol concentrations (Gregory et al. 2019), and the different groups will need to compete for this valuable electron donor. In our experiments, however, the limiting factor seems to be CO_2_/carbonate. When both were depleted or almost depleted, the H_2_ consuming activity also seized. Re-addition of CO_2_, led to an immediate restart of activity. This shows that there might be certain CO_2_ thresholds or CO_2_/H_2_ ratios, which directly control the microbial activity of the different microbial groups. Due to the fact that in our experiments the initial CO_2_/H_2_ ratios varied, it would be important for future studies to systematically test starting ratios in presence of a mixed community and carefully follow consumption and community development similar to Lafont et al. (Lafont et al. 2026). When rock material was added, we observed a general increase in activity, probably due to several factors: (I) a buffering in pH; (II) the rock contains carbonates, which can be used; (III) the rock contained acetogenic spores, which resumed activity when in contact with the brine, and this increased the cell numbers present; (IV) and the rock surface enhances the growth of sessile microbes (biofilm) (Dong et al. 2022).

The addition of original rock drastically increased the H_2_/CO_2_ ratios (Table 3), which we attribute to the fact that the rock provided an additional CO_2_/carbonate source for the microbes, and much higher methane and acetate yields were obtained. We expected initially to have the pH stabilized by the rock material, but contrary to the brine with rock ended with the highest pH values reaching over pH 10. Apparently, the increased use of CO_2_/carbonate and additional consumption of protons necessary for the metabolism led to this strong pH increase without an apparent buffering. We could also conclude that the rock material itself contains acetogenic spores. Autoclaving the brine and the rock resulted in the deactivation of SRB and methanogens, but acetate/formate production was still observed. Triple autoclavation was not sufficient to kill the spores, which underlines the difficulty of sterilizing original rock (Bartak et al. 2025). Overall, the presence of rock minerals has a positive effect on microbes and will lead to an increased H_2_ consumption, also by the fact that the rock might still carry additional H_2_-consuming microbes even after decades of dry and oxic storage. Unfortunately, we were not able to identify the acetogens, but it would be very interesting to perform further DNA extraction optimization from the rock to identify the microbes.

## Conclusions

The investigated reservoir brine from Austria contains a divers H_2_ consuming community, and we observed the activity of sulphate-reducing microbes, methanogens, and acetogens. During growth on H_2_, the community was mainly limited in CO_2_/carbonate content and could be continuously stimulated if CO_2_ was provided repeatedly. The presence of rock material additionally boosted microbial activity by providing additional carbonate and potentially introducing long lasting acetogenic spores. Our study shows that a complex community, which is oriented around H_2_ and CO_2_ occurrence, will compete over both substrates, leading to a co-current H_2_ consumption over time. Further work including more in-depth studies using isotopically labeled carbon sources and careful combination with enumeration of metabolic genes are needed to illuminate the true role of the different metabolisms and get the full carbon-balance. Overall, this study shows that careful evaluation of microbial activity prior to field tests can help to pinpoint the most suitable reservoirs and increase understanding of governing growth factors.

## Supplementary Material

fnag031_Supplemental_File

## Data Availability

All main data generated or analyzed during this study are included in this published article (and its Supplementary material files). Sequences are available online.
