# Supplementary material for: Hydrogen competition between a gas reservoir community indicates available CO2 as main limiting factor
Source: FEMS Microbiol Lett. 2026 Mar 24;373:fnag031. doi: 10.1093/femsle/fnag031 (PMC13070560; doi:10.1093/femsle/fnag031)
Supplement: fnag031_Supplemental_File [file fnag031_supplemental_file.docx]

SUPPLEMENTAL INFORMATION

Hydrogen competition between a gas reservoir community indicates available CO_2_ as main limiting factor

Nicole Dopffel^1*^, Larissa Compassi^2^, Ben Heydolph^1^, Abduljelil Kedir^1^, Biwen Annie An-Stepec^1^, Johann Badstöber^2^, Tzvetanka Iordanova Boiadjieva-Scherzer^2^

^1^ NORCE Research AS, Norway

^2^ OMV E&P GmbH, Austria

*To whom correspondence should be sent:

Nicole Dopffel, - NORCE

Nygårdsgaten 112, 5008 Bergen, Norway

Phone: +47 56 10 71 51

Email: nicd@norceresearch.no

Table S1. Mineralogical composition by XRD core analysis.

| Crushed core size | Unit | 2.5-5mm | 2.5mm | 2.5-5mm |
| --- | --- | --- | --- | --- |
| Depth | m | 669.60 | 669.60 | 669.50 |
| Quartz | mass % | 38.8 | 55.5 | 21.6 |
| Calcite | mass % | 39.8 | 25.9 | 49.5 |
| K-Fsp. | mass % | 18.0 | 14.1 | 24.9 |
| Plag | mass % | 1.7 | 1.9 | 1.9 |
| Dolomite | mass % | 0.4 | 0.7 | 0.4 |
| Clay Tot+Mica | mass % | 0.9 | 0.9 | 0.3 |
| Ankerite | mass % | 0.4 | 0.7 | 1.1 |
| Pyrite | mass % | - | 0.4 | 0.2 |

Table S2. Reads, observed ASVs and diversity index of the successfully sequenced samples. Only samples with more than 1000 reads were considered.

| **Identity** | **Sample Nr** | **Reads** | **Observed ASVs** | **Simpson** | **Shannon** |
| --- | --- | --- | --- | --- | --- |
| Original brine | sample_001 | 31637 | 58 | 0.0791 | 2.88 |
| Original brine | sample_002 | 23726 | 55 | 0.118 | 2.68 |
| End experiment with 95% H2 | sample_004 | 28153 | 45 | 0.133 | 2.46 |
| End experiment with 10% H2 | sample_006 | 31689 | 40 | 0.222 | 1.97 |
| End experiment with 10% H2 | sample_007 | 34663 | 43 | 0.284 | 1.89 |

Table S3. pH values over time in days measured in the bottles of the growth experiment, with the addition of different H_2_/CO_2_ ratios

| **Additions** | **Initial H2/CO2 ratio** | **0** | **7** | **13** | **21** | **30** | **37** | **50** | **72** | **93** |
| --- | --- | --- | --- | --- | --- | --- | --- | --- | --- | --- |
| +95% H2/CO2 | 12.1 | 7.4 | 7.8 | 7.8 | 7.9 | 8 | 7.9 | 8.2 | 8.4 | 8.1 |
| +95% H2/CO2 | 11.9 | 7.4 | 7.7 | 7.8 | 7.9 | 8.1 | 8 | 8.2 | 8.3 | 7.9 |
| + 40%H2/CO2 | 30.6 | 7.9 | 8.1 | 8.4 | 8.5 | 8.8 | 8.7 | 8.9 | 8.9 | 8.8 |
| + 40%H2/CO2 | 25.7 | 7.9 | 8.1 | 8.3 | 8.3 | 8.8 | 9.1 | 9.2 | 9.3 | 8.8 |
| + 10% H2/CO2 | 6.6 | 7.8 | 8 | 8.2 | 8 | 8.4 | 7.8 | 8.1 | 8.1 | 8.4 |
| + 10% H2/CO2 | 7.9 | 7.8 | 8.1 | 8.2 | 8.4 | 8.5 | 7.9 | 8 | 8.4 | 7.9 |
| +100 N2 | - | 7.8 | 7.8 | 8 | 7.8 | 8 | 8 | 8 | 7.9 | 7.7 |
| +100 N2 | - | 7.8 | 7.8 | 7.9 | 7.9 | 8 | 8 | 7.9 | 7.9 | 7.9 |
| STERILE + 10% H2/CO2 41C | 5.1 | 7.8 | 7.8 | 7.8 | 7.8 |  |  | 7.9 |  |  |
| STERILE + 10% H2/CO2 41C | 5.0 | 7.8 | 7.8 | 7.8 | 7.9 |  |  | 7.9 |  |  |

Table S4. Acetic acid values [mmol/L] over time in days, measured in the bottles of the growth experiment with the addition of different H_2_/CO_2_ ratios

| Sample | **d0** | **d7** | **d14** | **d21** | **d31** | **d51** | **d72** | **d93** |
| --- | --- | --- | --- | --- | --- | --- | --- | --- |
| +95% H2/CO2 | 4.93 | 4.92 | 4.88 | 5.02 | 5.09 | 5.21 | 5.19 | 5.46 |
| +95% H2/CO2 | 4.93 | 4.98 | 5.13 | 6.05 | 6.82 | 7.64 | 7.91 | 0.94 |
| + 40%H2/CO2 | 4.93 |  | 4.94 | 4.90 | 5.12 | 5.25 | 5.14 | 5.49 |
| + 40%H2/CO2 | 4.93 | 4.93 | 4.99 | 6.27 | 6.18 | 6.41 | 6.27 | 7.04 |
| + 10% H2/CO2 | 4.93 | 5.01 | 4.90 | 5.05 | 5.07 | 5.12 | 5.26 | 5.37 |
| + 10% H2/CO2 | 4.93 | 4.93 | 4.96 | 5.03 | 5.07 | 5.10 | 5.09 | 5.08 |
| +100N2 | 4.93 | 5.02 | 4.99 | 4.97 | 5.10 | 5.27 | 5.54 |  |
| +100N2 | 4.93 | 4.97 | 5.06 | 5.00 | 4.99 | 5.17 | 5.33 |  |

Table S5. Formic acid values [mmol/L] over time in days measured in the bottles of the growth experiment with the addition of different H_2_/CO_2_ ratios

|  | **0** | **7** | **14** | **21** | **31** | **51** | **72** | **93** |
| --- | --- | --- | --- | --- | --- | --- | --- | --- |
| +95% H2/CO2 | 0.00 | 0.13 | 0.25 | 0.34 | 0.43 | 1.11 | 1.09 | 0.76 |
| +95% H2/CO2 | 0.00 | 0.31 | 0.56 | 0.85 | 1.01 | 1.21 | 1.44 | 1.57 |
| + 40%H2/CO2 | 0.00 | 0.01 | 0.03 | 0.09 | 0.19 | 0.17 | 0.19 | 0.10 |
| + 40%H2/CO2 | 0.00 | 0.00 | 0.18 | 0.81 | 0.89 | 0.88 | 0.90 | 1.06 |
| + 10% H2/CO2 | 0.00 | 0.00 | 0.11 | 0.17 | 0.00 | 0.00 | 0.13 | 0.10 |
| + 10% H2/CO2 | 0.00 | 0.01 | 0.33 | 0.03 | 0.00 | 0.00 | 0.27 | 0.00 |
| +100N2 | 0 | 0 | 0 | 0 | 0 | 0 | 0 |  |
| +100N2 | 0 | 0 | 0 | 0 | 0 | 0 | 0 |  |

Table S6. pH values overtime in days measured in the bottles of the experiment with added minerals

|  | **-4** | **-1** | **0** | **7** | **14** | **21** | **28** | **35** |
| --- | --- | --- | --- | --- | --- | --- | --- | --- |
| w/o minerals | 7.9 | 7.7 | 7.6 | 8.2 | 8.4 | 8.4 | 8.7 | 9 |
| w/o minerals | 8 | 7.7 | 7.7 | 8.2 | 8.4 | 8.5 | 8.9 | 9 |
| autoclaved 10% minerals | 8.9 | 8 | 7.9 | 8.2 | 8.2 | 8.1 | 8 | 8.3 |
| autoclaved 10% minerals | 8.9 | 8.1 | 7.8 | 8 | 8.2 | 8 | 8.1 | 8.2 |
| autoclaved 50% minerals | 8.7 | 7.8 | 7.7 | 7.9 | 8.1 | 7.7 | 8 | 8.2 |
| autoclaved 50% minerals | 8.7 | 7.8 | 7.7 | 8.5 | 8.6 | 8.6 | 8.6 | 8.8 |
| +10% minerals | 7.8 | 7.6 | 7.6 | 8.4 | 9.1 | 8.9 | 9.5 | 9.7 |
| +10% minerals | 7.9 | 7.4 | 7.7 | 8.2 | 8.2 | 8.5 | 8.5 | 8.9 |
| +50% minerals | 7.9 | 7.2 | 7.6 | 8.3 | 8.7 | 8.9 | 9.4 | 9.9 |
| +50% minerals | 7.8 | 7.4 | 7.6 | 8.3 | 8.8 | 8.8 | 9.4 | 10.1 |

Table S7. Acetic acid values [mmol/L] over time in days in the bottles of the experiment with added minerals

| **Sample** | **d0** | **d7** | **d14** | **d21** | **d28** | **d35** |
| --- | --- | --- | --- | --- | --- | --- |
| w/o minerals | 4.74 | 5.05 | 5.06 | 5.67 | 5.89 | 5.92 |
| w/o minerals | 4.77 | 4.92 | 5.03 | 5.28 | 5.64 | 5.66 |
| autoclaved 10% minerals | 5.18 | 5.00 | 4.98 | 5.03 | 5.08 | 5.08 |
| autoclaved 10% minerals | 4.94 | 4.96 | 4.95 | 5.04 | 5.05 | 5.10 |
| autoclaved 50% minerals | 5.08 | 4.99 | 5.26 | 5.26 | 5.72 | 6.48 |
| autoclaved 50% minerals | 5.22 | 4.99 | 5.05 | 5.12 | 5.13 | 5.14 |
| +10% minerals | 4.77 | 5.31 | 7.14 | 7.63 | 7.73 | 7.45 |
| +10% minerals | 4.77 | 4.94 | 6.20 | 6.95 | 6.82 | 6.82 |
| +50% minerals | 4.76 | 4.83 | 6.33 | 7.60 | 7.92 | 7.94 |
| +50% minerals | 4.84 | 5.61 | 7.74 | 8.90 | 9.10 | 9.14 |

Table S8. Acetic acid values [mmol/L] over time in days in the bottles of the experiment with added minerals, which have been autoclaved three times on three consecutive days.

| **Sample** | **d0** | **d14** | **d20** | **d27** | **d34** | **d45** |
| --- | --- | --- | --- | --- | --- | --- |
| Only brine with 10% H2 | 0.02 | 0.11 | 0.39 | 0.62 | 0.78 | 1.12 |
| Brine plus 20 g sediment 10% H2 | 0.09 | 0.05 | 0.07 | 0.09 | 0.08 | 0.16 |
| Brine plus 20 g sediment 10% H2 | 0.09 | 0.06 | 0.08 | 0.11 | 0.29 | 0.45 |
| Autoclaved brine 10% H2 | 0.32 | 0.35 | 0.33 | 0.34 | 0.33 | 0.34 |
| Autoclaved brine 10% H2 | 0.32 | 0.33 | 0.34 | 0.35 | 0.36 | 0.33 |
| 3x autoclaved sediment plus autoclaved brine 10% H2 | 0.09 | 0.19 | 0.60 | 2.06 | 2.72 | 3.69 |
| 3x autoclaved sediment plus autoclaved brine 10% H2 | 0.09 | 0.15 | 0.55 | 1.92 | 2.67 | 3.55 |

Table S9: H_2_ and CO_2_ [mmol] over time in days in the bottles of the experiment with added minerals, which have been autoclaved three times on three consecutive days.

|  | 0 | 14 | 20 | 27 | 34 | 45 |
| --- | --- | --- | --- | --- | --- | --- |
|  | H_2_ | | | | | |
| Only brine with 10% H2 | 0.63 | 0.58 | 0.51 | 0.42 | 0.42 | 0.42 |
| Brine plus 20 g sediment 10% H2 | 0.57 | 0.26 | 0.25 | 0.25 | 0.13 | 0.14 |
| Brine plus 20 g sediment 10% H2 | 0.59 | 0.19 | 0.18 | 0.17 | 0.11 | 0.11 |
| Autoclaved brine 10% H2 | 0.61 | 0.58 | 0.57 | 0.57 | 0.56 | 0.55 |
| Autoclaved brine 10% H2 | 0.68 | 0.66 | 0.65 | 0.64 | 0.64 | 0.63 |
| 3x autoclaved sediment plus autoclaved brine 10% H2 | 0.60 | 0.54 | 0.49 | 0.37 | 0.32 | 0.26 |
| 3x autoclaved sediment plus autoclaved brine 10% H2 | 0.63 | 0.57 | 0.53 | 0.41 | 0.35 | 0.29 |
|  | CO_2_ | | | | | |
| Only brine with 10% H2 | 0.05 | 0.05 | 0.04 | 0.03 | 0.02 | 0.02 |
| Brine plus 20 g sediment 10% H2 | 0.02 | 0.01 | 0.01 | 0.01 | 0.00 | 0.01 |
| Brine plus 20 g sediment 10% H2 | 0.06 | 0.01 | 0.01 | 0.01 | 0.01 | 0.01 |
| Autoclaved brine 10% H2 | 0.07 | 0.07 | 0.07 | 0.07 | 0.07 | 0.07 |
| Autoclaved brine 10% H2 | 0.09 | 0.08 | 0.08 | 0.08 | 0.08 | 0.08 |
| 3x autoclaved sediment plus autoclaved brine 10% H2 | 0.10 | 0.12 | 0.11 | 0.08 | 0.07 | 0.05 |
| 3x autoclaved sediment plus autoclaved brine 10% H2 | 0.10 | 0.13 | 0.11 | 0.08 | 0.07 | 0.05 |

Table S10: Relative abundance of the sequenced samples including all ASVs and closest identity

| **Initial average** | with 95% H2 | with 10% H2 | with 10% H2 | Id |
| --- | --- | --- | --- | --- |
| 10.02 | 26.70 | 11.60 | 9.31 | d:Bacteria(1.0000),p:Desulfobacterota(1.0000),c:Desulfovibrionia(1.0000),o:Desulfovibrionales(1.0000),f:Desulfovibrionaceae(1.0000),g:Desulfovibrio(0.9700),s:uncultured_bacterium(0.8536) |
| 6.23 | 15.50 | 5.19 | 4.17 | d:Bacteria(1.0000),p:Desulfobacterota(1.0000),c:Desulfovibrionia(1.0000),o:Desulfovibrionales(1.0000),f:Desulfovibrionaceae(1.0000),g:Desulfovibrio(0.9500),s:uncultured_bacterium(0.6840) |
| 2.91 | 14.20 | 10.90 | 1.06 | d:Archaea(1.0000),p:Euryarchaeota(1.0000),c:Methanobacteria(1.0000),o:Methanobacteriales(1.0000),f:Methanobacteriaceae(1.0000),g:Methanobacterium(1.0000),s:Methanobacterium_formicicum(0.7700) |
| 0.10 | 7.48 | 42.10 | 10.20 | d:Bacteria(1.0000),p:Firmicutes(1.0000),c:Moorellia(1.0000),o:Desulfitibacterales(1.0000),f:Desulfitibacteraceae(1.0000),g:Desulfitibacter(1.0000),s:uncultured_Firmicutes_bacterium(1.0000) |
| 20.95 | 6.78 | 2.31 | 4.77 | d:Bacteria(1.0000),p:Proteobacteria(1.0000),c:Alphaproteobacteria(1.0000),o:Rhodospirillales(1.0000),f:Rhodospirillaceae(1.0000),g:Candidatus_Riegeria(1.0000),s:uncultured_bacterium(0.8800) |
| 0.01 | 4.29 | 0.65 | 1.35 | d:Bacteria(1.0000),p:Firmicutes(1.0000),c:Clostridia(1.0000),o:Peptostreptococcales-Tissierellales(1.0000),f:Family_XI(1.0000),g:Soehngenia(0.9600),s:uncultured_bacterium(0.8448) |
| 0.06 | 3.85 | 8.05 | 4.42 | d:Bacteria(1.0000),p:Firmicutes(1.0000),c:Clostridia(0.8800),o:Peptostreptococcales-Tissierellales(0.7744),f:Family_XI(0.6815),g:Soehngenia(0.5997),s:uncultured_bacterium(0.2759) |
| 6.94 | 3.40 | 0.10 | 0.46 | d:Bacteria(1.0000),p:Desulfobacterota(1.0000),c:Desulfovibrionia(1.0000),o:Desulfovibrionales(1.0000),f:Desulfovibrionaceae(1.0000),g:Desulfovibrio(1.0000),s:Desulfovibrio_sp._ds2-2(0.4500) |
| 0.05 | 3.29 | 7.98 | 4.45 | d:Bacteria(1.0000),p:Firmicutes(1.0000),c:Clostridia(1.0000),o:Peptostreptococcales-Tissierellales(1.0000),f:Family_XI(1.0000),g:Soehngenia(1.0000),s:uncultured_Soehngenia_sp.(0.3500) |
| 3.05 | 2.36 | 5.72 | 0.03 | d:Bacteria(1.0000),p:Firmicutes(1.0000),c:Clostridia(1.0000),o:Caldicoprobacterales(1.0000),f:Caldicoprobacteraceae(1.0000),g:Caldicoprobacter(1.0000),s:uncultured_bacterium(1.0000) |
| 0.12 | 1.92 | 0.01 | 0.00 | d:Bacteria(1.0000),p:Firmicutes(1.0000),c:Thermovenabulia(1.0000),o:Thermovenabulales(1.0000),g:Tepidanaerobacter(1.0000),s:Thermoanaerobacteraceae_bacterium_HZ254T(0.9600) |
| 5.11 | 1.64 | 0.21 | 0.86 | d:Bacteria(1.0000),p:Desulfobacterota(1.0000),c:Desulfovibrionia(1.0000),o:Desulfovibrionales(1.0000),f:Desulfovibrionaceae(1.0000),g:Desulfovibrio(1.0000),s:Desulfovibrio_desulfuricans(0.2800) |
| 0.02 | 1.50 | 0.01 | 0.00 | d:Bacteria(1.0000),p:Firmicutes(1.0000),c:Moorellia(0.9900),o:Moorellales(0.9702),f:Moorellaceae(0.9508),g:Moorella(0.9318),s:uncultured_Moorella_sp.(0.5404) |
| 0.00 | 1.03 | 0.00 | 0.00 | d:Archaea(1.0000),p:Halobacterota(0.9700),c:Methanomicrobia(0.9409),o:Methanomicrobiales(0.9127),f:Methanomicrobiaceae(0.8762),g:Methanoculleus(0.8411),s:uncultured_archaeon(0.3701) |
| 0.09 | 0.93 | 1.34 | 1.89 | d:Bacteria(1.0000),p:Thermotogota(1.0000),c:Thermotogae(1.0000),o:Petrotogales(1.0000),f:Petrotogaceae(1.0000),g:Defluviitoga(1.0000),s:uncultured_Thermotogaceae_bacterium(0.9900) |
| 0.35 | 0.81 | 0.01 | 0.01 | d:Archaea(1.0000),p:Thermoplasmatota(1.0000),c:Thermoplasmata(1.0000),o:Methanomassiliicoccales(1.0000),f:Methanomassiliicoccaceae(1.0000),g:Methanomassiliicoccus(1.0000),s:uncultured_archaeon(0.7400) |
| 0.26 | 0.60 | 0.08 | 0.12 | d:Bacteria(1.0000),p:Bacteroidota(1.0000),c:Bacteroidia(1.0000),o:Bacteroidales(1.0000),f:Dysgonomonadaceae(0.9800),g:Proteiniphilum(0.9506),s:uncultured_Bacteroidetes_bacterium(0.3802) |
| 0.32 | 0.48 | 0.00 | 0.00 | d:Bacteria(1.0000),p:Firmicutes(1.0000),c:Thermovenabulia(1.0000),o:Thermovenabulales(1.0000),g:Tepidanaerobacter(1.0000),s:Thermoanaerobacterales_bacterium_SK-G1(0.4400) |
| 0.46 | 0.43 | 0.01 | 0.14 | d:Bacteria(1.0000),p:Spirochaetota(1.0000),c:Spirochaetia(1.0000),o:Spirochaetales(1.0000),f:Spirochaetaceae(1.0000),g:uncultured(1.0000),s:Rectinema_cohabitans(0.6300) |
| 0.02 | 0.41 | 0.00 | 0.02 | d:Bacteria(1.0000),p:Desulfobacterota(0.8400),c:Desulfovibrionia(0.7056),o:Desulfovibrionales(0.5927),f:Desulfovibrionaceae(0.4979),g:Desulfovibrio(0.4182),s:Desulfovibrio_alaskensis_G20(0.3262) |
| 0.59 | 0.29 | 0.65 | 0.11 | d:Bacteria(1.0000),p:Synergistota(1.0000),c:Synergistia(1.0000),o:Synergistales(1.0000),f:Synergistaceae(1.0000),g:Aminiphilus(1.0000),s:Aminiphilus_circumscriptus(0.7000) |
| 1.02 | 0.29 | 0.04 | 0.07 | d:Bacteria(1.0000),p:Firmicutes(1.0000),c:Clostridia(1.0000),o:Eubacteriales(1.0000),f:Alkalibacteraceae(1.0000),g:Alkalibacter(1.0000),s:uncultured_bacterium(0.9200) |
| 0.30 | 0.24 | 0.15 | 0.22 | d:Bacteria(1.0000),p:Bacteroidota(1.0000),c:Bacteroidia(1.0000),o:Bacteroidales(1.0000),f:Dysgonomonadaceae(1.0000),g:Proteiniphilum(1.0000),s:uncultured_bacterium(0.8300) |
| 3.02 | 0.19 | 0.04 | 0.10 | d:Bacteria(1.0000),p:Firmicutes(1.0000),c:Desulfitobacteriia(1.0000),o:Desulfitobacteriales(1.0000),s:Desulfosporosinus_sp._FE18(0.9600) |
| 1.87 | 0.18 | 0.02 | 0.04 | d:Bacteria(1.0000),p:Firmicutes(1.0000),c:Clostridia(0.9600),o:Peptococcales(0.8928),f:Peptococcaceae(0.8303),g:uncultured(0.7722),s:uncultured_bacterium(0.6718) |
| 1.49 | 0.17 | 0.01 | 0.01 | d:Bacteria(1.0000),p:Chloroflexi(1.0000),c:Anaerolineae(1.0000),o:Anaerolineales(1.0000),f:Anaerolineaceae(1.0000),g:uncultured(0.8200),s:uncultured_bacterium(0.8036) |
| 4.65 | 0.17 | 0.10 | 0.31 | d:Bacteria(1.0000),p:Firmicutes(1.0000),c:Clostridia(1.0000),o:Eubacteriales(1.0000),f:Eubacteriaceae(1.0000),g:Acetobacterium(1.0000),s:uncultured_Firmicutes_bacterium(0.5700) |
| 0.12 | 0.14 | 0.00 | 0.01 | d:Bacteria(1.0000),p:Firmicutes(0.9500),c:Moorellia(0.5130),o:NRB23(0.1283),s:uncultured_bacterium(0.0782) |
| 11.75 | 0.12 | 0.06 | 0.12 | d:Bacteria(1.0000),p:Desulfobacterota(1.0000),c:Desulfuromonadia(1.0000),o:Desulfuromonadales(1.0000),f:Desulfuromonadaceae(1.0000),s:uncultured_bacterium(0.5000) |
| 8.74 | 0.12 | 0.07 | 0.25 | d:Bacteria(1.0000),p:Firmicutes(1.0000),c:Moorellia(1.0000),o:Moorellales(1.0000),f:Moorellaceae(1.0000),g:Moorella(1.0000),s:uncultured_Clostridia_bacterium(0.8000) |
| 0.81 | 0.07 | 0.02 | 0.03 | d:Bacteria(1.0000),p:Firmicutes(1.0000),c:Desulfitobacteriia(1.0000),o:Desulfitobacteriales(1.0000),s:Desulfosporosinus_sp._FE18(0.9600) |
| 0.03 | 0.07 | 0.39 | 0.31 | d:Bacteria(1.0000),p:Firmicutes(1.0000),c:Bacilli(1.0000),o:Bacillales(1.0000),f:Bacillaceae(0.9100),g:Tepidibacillus(0.4641),s:Tepidibacillus_infernus(0.2135) |
| 0.80 | 0.06 | 0.01 | 0.04 | d:Bacteria(1.0000),p:Firmicutes(1.0000),c:uncultured(1.0000),s:uncultured_bacterium(1.0000) |
| 0.24 | 0.06 | 0.06 | 0.00 | d:Bacteria(1.0000),p:Thermotogota(1.0000),c:Thermotogae(1.0000),o:Kosmotogales(1.0000),f:Kosmotogaceae(1.0000),g:Mesotoga(1.0000),s:uncultured_bacterium(0.8700) |
| 0.98 | 0.04 | 0.01 | 0.04 | d:Bacteria(1.0000),p:Firmicutes(1.0000),c:Clostridia(1.0000),o:Clostridiales(1.0000),f:Clostridiaceae(1.0000),g:Clostridium_sensu_stricto_7(1.0000),s:Clostridium_homopropionicum(0.5000) |
| 0.22 | 0.04 | 0.00 | 0.02 | d:Bacteria(1.0000),p:Firmicutes(1.0000),c:Desulfotomaculia(1.0000),o:Desulfotomaculales(1.0000),f:Desulfotomaculaceae(1.0000),g:Desulfotomaculum(1.0000),s:Desulfotomaculum_reducens_MI-1(0.4300) |
| 0.93 | 0.03 | 0.02 | 0.07 | d:Bacteria(1.0000),p:Firmicutes(0.9900),c:Clostridia(0.5940),o:Thermincolales(0.1010),f:Thermincolaceae(0.0172),g:Thermincola(0.0029),s:uncultured_bacterium(0.0022) |
| 0.18 | 0.03 | 0.00 | 0.00 | d:Bacteria(1.0000),p:Desulfobacterota(1.0000),c:Desulfovibrionia(1.0000),o:Desulfovibrionales(1.0000),f:Desulfovibrionaceae(1.0000),g:Desulfovibrio(0.9300),s:Desulfovibrio_vulgaris_str._Miyazaki_F(0.2604) |
| 0.05 | 0.01 | 0.00 | 0.01 | d:Bacteria(1.0000),p:Firmicutes(1.0000),c:Clostridia(1.0000),o:Caldicoprobacterales(1.0000),f:Caldicoprobacteraceae(1.0000),g:Caldicoprobacter(1.0000),s:uncultured_bacterium(0.8000) |
| 0.44 | 0.01 | 0.00 | 0.03 | d:Bacteria(1.0000),p:Firmicutes(1.0000),c:Clostridia(1.0000),o:Lachnospirales(1.0000),f:Lachnospiraceae(1.0000),g:Lachnoclostridium(0.9100),s:Clostridium_methoxybenzovorans_SR3(0.1547) |
| 0.27 | 0.01 | 0.01 | 0.00 | d:Bacteria(1.0000),p:Firmicutes(1.0000),c:Incertae_Sedis(1.0000),o:DTU014(1.0000),s:uncultured_bacterium(0.9200) |
| 0.23 | 0.01 | 0.00 | 0.00 | d:Bacteria(1.0000),p:Firmicutes(1.0000),c:Incertae_Sedis(1.0000),o:DTU014(1.0000),s:uncultured_bacterium(0.9900) |
| 0.21 | 0.01 | 0.00 | 0.00 | d:Bacteria(1.0000),p:Firmicutes(1.0000),c:uncultured(1.0000),s:uncultured_bacterium(1.0000) |
| 2.90 | 0.00 | 0.00 | 0.00 | d:Bacteria(1.0000),p:Firmicutes(1.0000),c:Desulfotomaculia(0.9900),o:Desulfotomaculales(0.9801),f:Desulfurisporaceae(0.9703),g:SCADC1-2-3(0.9315),s:uncultured_bacterium(0.8570) |
| 0.04 | 0.00 | 0.00 | 0.00 | d:Bacteria(1.0000),p:Synergistota(0.9800),c:Synergistia(0.9604),o:Synergistales(0.9412),f:Synergistaceae(0.9224),g:Aminivibrio(0.8209),s:Aminivibrio_pyruvatiphilus(0.4679) |
| 0.32 | 0.00 | 0.00 | 0.00 | d:Bacteria(1.0000),p:Firmicutes(1.0000),c:Clostridia(1.0000),o:Eubacteriales(0.9800),f:Eubacteriaceae(0.9604),g:Eubacterium(0.9316),s:Eubacterium_callanderi(0.2049) |
| 0.31 | 0.00 | 0.00 | 0.00 | d:Bacteria(1.0000),p:Firmicutes(1.0000),c:Desulfotomaculia(1.0000),o:Desulfotomaculales(1.0000),f:Pelotomaculaceae(1.0000),g:uncultured(0.4900),s:uncultured_bacterium(0.3038) |
| 0.14 | 0.00 | 0.00 | 0.00 | d:Bacteria(1.0000),p:Synergistota(1.0000),c:Synergistia(1.0000),o:Synergistales(1.0000),f:Synergistaceae(1.0000),g:Thermovirga(1.0000),s:uncultured_Synergistetes_bacterium(0.7800) |
| 0.10 | 0.00 | 0.00 | 50.50 | d:Bacteria(1.0000),p:Firmicutes(1.0000),c:Clostridia(1.0000),o:Peptostreptococcales-Tissierellales(1.0000),f:Thermotaleaceae(1.0000),g:Geosporobacter(0.9900),s:Geosporobacter_ferrireducens(0.8910) |
| 0.09 | 0.00 | 0.00 | 0.00 | d:Bacteria(1.0000),p:Desulfobacterota(1.0000),c:Desulfobacteria(1.0000),o:Desulfobacterales(1.0000),f:Desulfobacteraceae(1.0000),g:Desulfobotulus(1.0000),s:Desulfobotulus_sapovorans(1.0000) |
| 0.02 | 0.00 | 0.18 | 0.17 | d:Bacteria(1.0000),p:Firmicutes(1.0000),c:Clostridia(1.0000),o:Peptostreptococcales-Tissierellales(1.0000),g:Clostridium_sensu_stricto(0.9200),s:Clostridium_formicaceticum(0.6624) |
| 0.02 | 0.00 | 0.00 | 0.00 | d:Bacteria(1.0000),p:Firmicutes(1.0000),c:Clostridia(0.9500),o:Oscillospirales(0.9025),f:Hungateiclostridiaceae(0.8574),g:Ruminiclostridium(0.7802),s:uncultured_bacterium(0.5539) |
| 0.02 | 0.00 | 0.00 | 0.00 | d:Bacteria(1.0000),p:Firmicutes(1.0000),c:Clostridia(0.9800),o:Christensenellales(0.9506),f:Christensenellaceae(0.9221),g:uncultured(0.8944),s:uncultured_bacterium(0.5545) |
| 0.01 | 0.00 | 1.92 | 4.24 | d:Archaea(1.0000),p:Euryarchaeota(1.0000),c:Methanobacteria(1.0000),o:Methanobacteriales(1.0000),f:Methanobacteriaceae(1.0000),g:Methanobacterium(0.8300),s:Methanobacterium_alcaliphilum(0.1660) |
